# Effect of Ultra-High-Pressure Treatment on *Gastrodia elata* Blume: Drying Characteristics, Components, and Neuroprotective Activity

**DOI:** 10.3390/foods13223534

**Published:** 2024-11-05

**Authors:** Hongjing Dong, Shuang Liu, Xinming Wang, Meng Li, Buddhika Niroshie Perumpuli Arachchige, Xiao Wang

**Affiliations:** 1 Key Laboratory for Applied Technology of Sophisticated Analytical Instruments of Shandong Province, Shandong Analysis and Test Center, Qilu University of Technology (Shandong Academy of Sciences), Jinan 250014, Chinabuddhikap@fst.ruh.ac.lk (B.N.P.A.); 2 Key Laboratory for Natural Active Pharmaceutical Constituents Research in Universities of Shandong Province, School of Pharmaceutical Sciences, Qilu University of Technology (Shandong Academy of Sciences), Jinan 250014, China

**Keywords:** *Gastrodia elata* blume, ultra-high pressure, hot air drying, quality enhancement

## Abstract

Gastrodiae Rhizoma (GE), a popular food in China, is stored and consumed after steaming, which can lead to the degradation of active substances and a decrease in its quality. Therefore, this study explored the potential application of ultra-high-pressure (UHP)-assisted hot air drying in improving the quality of GE. The results indicated that UHP pre-treatment could preserve the original cross-sectional color of GE and increase the degrees of freedom of water in GE samples. Compared with traditional steaming pre-treatment (18 h), UHP pre-treatment at 500 MPa significantly shorted the time (10 h) required for the GE samples to reach drying equilibrium. Meanwhile, the UHP-assisted hot air drying method (60 °C) could reduce the activity of *β*-D-glucosidase and avoid the degradation of active substances. Finally, UHP pre-treatment improved the neuroprotective activity in vivo. Overall, UHP-assisted hot air drying could improve the quality of GE samples. This study provides a simple method for improving the quality of GE samples and offers a reference for subsequent research on the influence of UHP on GE.

## 1. Introduction

Gastrodiae Rhizoma (GE), the rhizome of the Orchidaceae plant *Gastrodia elata* Blume, is a traditional Chinese medicine with homologous medicinal and edible properties [1]. It is primarily found in China, South Korea, Japan, and other countries [2]. It has many biological functions, which enable its usage as the treatment of headaches, paralysis, rheumatism, and other neurological diseases [3], and these biological activities are closely related to the compounds present in GE, such as gastrodin, parishins, and p-hydroxybenzyl alcohol. In addition to these compounds, a large amount of water is available in fresh GE. This available water is important for enzyme activity and chemical reactions, whereas inapplicable water can lead to the germination and decay of GE [4,5,6]. Thus, before use, fresh GE is commonly steamed and dried at low temperatures to preserve certain biologically active compounds and enhance its storage capacity [7]. However, a high temperature (100 °C, 20 min) during the steaming process can lead to the degradation of active substances and reduce the quality of the GE samples [8,9]. Therefore, developing a method that could replace traditional steaming pre-treatment and prevent the degradation of active compounds in GE is urgently needed.

Recently, hot air drying, microwave drying, freeze drying, and vacuum drying have been used to remove water in GE [8,10]. Among these methods, hot air drying is the most common processing method due to its simple operation, low cost, and controlled process [11]. However, long drying times and high energy consumption are the main drawbacks of the hot air drying method. In order to address these limitations, the use of different pre-treatments prior to drying is considered an efficient strategy [12]. Ultra-high-pressure (UHP) is a non-heat pre-treatment method that is widely applied for many purposes, such as enzyme deactivation, extending storage and preservation time, and accelerated steaming [13,14,15,16]. Previous studies report that UHP improves the drying rate and increases bioactivities through cell deformation and increases in cell permeability [17,18,19]. Thus, UHP pre-treatment coupled with hot air drying could improve the drying efficiency while contributing to obtain high-quality GE.

To the best of our knowledge, no study has been conducted on improving the quality of GE through UHP pre-treatment combined with hot air drying. Therefore, the present study aimed to investigate the effects of UHP pre-treatment combined with the hot-air drying method on the properties of GE. Specifically, the color and texture of GE were evaluated through a personal subjective judgment method; drying characteristic analysis was performed using low-field nuclear magnetic resonance (LF-NMR); the activity of degrading enzymes was assessed using a parishin A enzymatic hydrolysis experiment; the content of the active substances was determined by HPLC; and neuroprotective activity was evaluated using a zebrafish model. This study provides a basis for improving the quality of GE properties.

## 2. Materials and Methods

### 2.1. Sample Preparation

Fresh GE were collected from a planting base in (Zhaotong, China). The samples with an average weight of 70.0 ± 5.0 g and a diameter of 50.0 ± 5.0 mm were selected. After washing, residual moisture from the surface of the samples was removed using filter paper. To determine the moisture content, the samples were dried using a hot air dryer at 105 °C until a constant weight was obtained, and the average moisture content of fresh GE was determined as 80.48 ± 0.85%. The moisture content was determined in triplicates.

### 2.2. Ultra-High-Pressure (UHP) Treatment

Fresh GE samples were placed in polyethylene bags and vacuum-sealed. The samples were then transferred to a UHP instrument (Huatai Senmiao, Tianjin, China), and the liquid level of the water in the pressure kettle was adjusted to a suitable height for UHP pre-treatment. The samples were treated under different pressures (400, 500, and 600 MPa) for 10 min and labeled UHP-400, UHP-500, and UHP-600, respectively. A steamed GE (steamed for 15 min) sample was also prepared as a control (ST-100 group). Fresh GE samples without pre-treatment were used as negative controls and labeled as the UT-0 group.

### 2.3. Drying Experiments

#### 2.3.1. Hot-Air Drying Treatment

To explore the effects of UHP on the drying characteristics of GE, drying experiments were performed as described previously, with minor modifications [20]. After UHP pre-treatment, the GE samples with different pressure treatments were cut into blocks of 2.0 ± 0.1 cm in radius, 6.0 ± 0.5 cm in height, 2.0 ± 0.1 cm in thickness, and 10.0 ± 0.5 g in weight. Three GE samples were prepared in parallel from each treatment group. The GE blocks in different groups were dried in a laboratory-scale hot air oven (Huafei electric heating equipment, Suzhou, China) at 1.0 m/s and at a temperature of 40, 60, or 80 °C separately. The relative humidity of the drying environment was controlled at 18–22%. During drying, the samples were weighted every 30 min for the first 2 h, every 60 min for the next 2 h, and finally, every 120 min for the later time. The drying treatment was stopped until the mass difference between the two consecutive weights was less than 0.1 mg. The moisture content (*MC*) and drying rate (*DR*) were calculated using Equations (1) and (2) as follows:

(1) $$\mathrm{MC}=\frac{W_{t}-W_{d}}{W_{d}}$$

(2) $$\mathrm{DR}=\frac{M_{i}-M_{t}}{t-i}$$

where *W_t_* (g) is the mass of the sample at time *t* (min); *W_d_* is the constant mass of the dried sample; and *M_i_* (g/g, d.b.) and *M_t_* (g/g, d.b.) are the moisture contents at times *i* and *t*, respectively.

#### 2.3.2. Low-Field Nuclear Magnetic Resonance (LF-NMR) Measurements

The transverse relaxation time (T_2_) and magnetic resonance imaging (MRI) measurements of NMR were measured using LF-NMR (MesoMR23-060H–I, Niumag Corp., Shanghai, China) at a resonance frequency for protons of 20 MHz and at a temperature of 35 °C according to a previously published study [10]. The GE samples were placed in cylindrical glass tubes with an outer diameter of 25 mm. The main parameters were set as follows: waiting time (TW), 2000.000 ms; echo time (TE), 0.200 ms; number of echoes (NECH), 18,000; number of scans (NS), 8. The inversion software, MultiExp Inv Analysis software BV6.00.12 (Niumag Analytical Instrument Co., Suzhou, China), was used for iterative analysis (100, 000 iterations) to obtain the T_2_ curve.

A multi-layer spin echo (MSE) sequence was used to collect the proton density image of the sample cross-section and low-field MRI images of the water state and its changes during the hot-air drying process of the GE mass after different treatments were obtained. The main parameters were set as follows: The layer thickness was 3.0 mm; section gap was 3.0 mm; TE was 20.000 ms; and TR was 500.000 ms.

### 2.4. Determination of Active Substances Content of GE

A previous study suggested that gastrodin, parishins, and p-hydroxybenzyl alcohol are the active components of GE [21]. Therefore, the contents of six active substances in GE were determined: gastrodin, p-hydroxybenzyl alcohol, parishin A, parishin B, parishin C, and parishin E. The active substances were analyzed using HPLC (Acchrom Tech, Beijing, China) equipped with a Symmetry C18 chromatographic column (4.6 mm × 250 mm, 5 µm; Waters, Milford, MA, USA). The mobile phase was acetonitrile (A) and 0.1% formic acid in water (B) with gradient elution: 0–10 min, 3%−10% A; 10–15 min, 10%–12% A; 15–25 min, 12%–18% A; and 25–40 min, 18%–18% A. The flow rate was 1.0 mL/min. The column temperature was set at 25 °C. The detection wavelength was 225 nm, and the injection volume was 20 μL.

The active substances in different groups of GE samples were extracted using 70% ethanol at a 1:10 (g:mL) solid–liquid ratio of material to liquid by reflux extraction (83.83 ± 0.36 °C). Extraction time (1 h) was calculated after boiling the samples in 70% ethanol. After extraction, the solution was filtered using the suction filtration method. Finally, the supernatant was retained and filtered with a microporous filter membrane (0.45 μm) for subsequent analysis.

### 2.5. Measurement of Activity of Enzymes

The *β*-D-glucosidase was one of the main degrading enzymes in GE. A previous study demonstrated that *β*-D-glucosidase could hydrolyze ester bonds, leading to the degradation of parishins and gastrodin [22]. Therefore, the *β*-D-glucosidase activity in GE samples subjected to different UHP pre-treatment conditions was studied separately. Firstly, GE pieces (10.0 g) in different groups were placed in a mortar containing 20 mL of pre-cooled (4 °C) Na_2_HPO_4_-NaH_2_PO_4_ buffer solution (0.05 M, pH = 6.0). Subsequently, 0.6 g of crosslinked polyvinylpyrrolidone (PVPP) was added and homogenized at 4 °C. The homogenate was transferred to a 50 mL centrifuge tube and kept at 4 °C for 2 h. Thereafter, the homogenate was concentrated at 10,000 r/min for 25 min at 4 °C, and the resultant supernatant was collected. The supernatant was then transferred into a dialysis bag with a molecular weight cutoff of 8–14 KDa and dialyzed against phosphate buffer solution (0.05 M, pH = 6.0) at 4 °C for 24 h. Finally, the residue (crude enzyme of GE) was obtained.

The crude enzyme solution (1.8 mL) and parishin A (PA, 0.2 mg/mL) solution (0.2 mL) of each sample were placed in a 5 mL centrifuge tube and mixed for 1 min as the test group. Simultaneously, 1.8 mL of Na_2_HPO_4_-NaH_2_PO_4_ buffer solution and 0.2 mL of PA solution were also vortexed for 1 min as the control group. In addition, 1.8 mL of crude enzyme solution and 0.2 mL of Na_2_HPO_4_-NaH_2_PO_4_ buffer solution were also vortically mixed for 1 min to serve as the blank group. Each group was incubated in a water bath at 40 °C for 2 h, and 1 mL of acetonitrile was added to stop the incubation, followed by mixing and centrifuging (8000 r/min for 10 min). One millimeter of the supernatant was dried and redissolved using 1 mL of deionized water, and the solution was filtered through a 0.45 μm membrane for HPLC analysis. The activity of *β*-D-glucosidase was calculated using Equation (3) as follows:

(3) $$\mathrm{Activity}\;\mathrm{of}\;\beta \text{-}D\text{-}\mathrm{glucosidase}\;(\%)=\frac{A_{control}-(A_{test}-A_{blank})}{A_{control}}\times l00\%$$

where the *A_control_* represent the peak area of PA in control group; *A_test_* represents the peak area of PA in test group, *A_blank_* represents the peak area and blank groups, respectively.

### 2.6. Neuroprotective Activity In Vivo

The animals used in this study were obtained from the Shandong Academy of Sciences’ Institute of Biology (Shandong, China), under permit number SYXK-2020-0015. Ethical approval was granted by the Biology Institute’s Animal Care and Ethics Committee (approval code: SWS202200916), ensuring adherence to both European Directive 2010/63/EU and the institute’s own ethical guidelines for laboratory animal care and use.

According to a previously published method [23], transgenic zebrafish (*Vmat*: *GFP*) were selected as experimental animals to study neuroprotective activity. Briefly, mature zebrafish were placed in a breeding tank with a 1:1 male and female ratio, and the fertilized eggs were collected the following day. The eggs were cultured in zebrafish culture water containing methylene blue solution and incubated at 28 °C in an incubator for 24 h. Afterward, the outer membrane of the embryo was removed by treatment with streptomyces protease (1 mg/mL). The zebrafish were then transferred to a 6-well plate with 30 fish per well. They were divided into three groups: the test group (treated with 75, 50, 25, and 12.5 μg/mL GE 70% ethanol extract in the UHP-500 group), the control group (treated with zebrafish culture water only), and the 1-Methyl-4-phenyl-1,2,3,6-tetrahydropyridine (MPTP)-treated group (treated with 50 μmol/L MPTP). Zebrafish breeding water was changed daily. Meanwhile, 0.003% (*w*/*v*) PTU was added to each well to inhibit melanin production in juvenile zebrafish. On the fifth day after fertilization, the zebrafish were anesthetized and photographed. The experiment was repeated three times, and data analysis was performed using Image-Pro Plus software 6.0.0.260. Neuroprotective activity was calculated using Equation (4) as follows:

(4) $$\mathrm{Neuroprotective}\;\mathrm{activity}\;(\%)=\frac{L_{i}-L_{j}}{L_{0}-L_{j}}\times l00\%$$

where *L_i_* is the dopamine neuron region length in the sample group, *L_0_* is the dopamine neuron region length in the blank group, and *L_j_* is the dopamine neuron region length in the model group.

### 2.7. Statistical Analysis

The data was presented as the mean ± SD of each measurement. Microsoft Office Excel and Origin 9 software were used to analyze the moisture content and LF-NMR data. Differences between the two groups were evaluated using analysis of variance (ANOVA). Statistical significance was set at *p* < 0.05. Differences were considered significant at *p* < 0.01, which indicated an extremely significant difference.

## 3. Results and Discussion

### 3.1. Color Analysis of UHP-Treated GE

**Figure 1:**
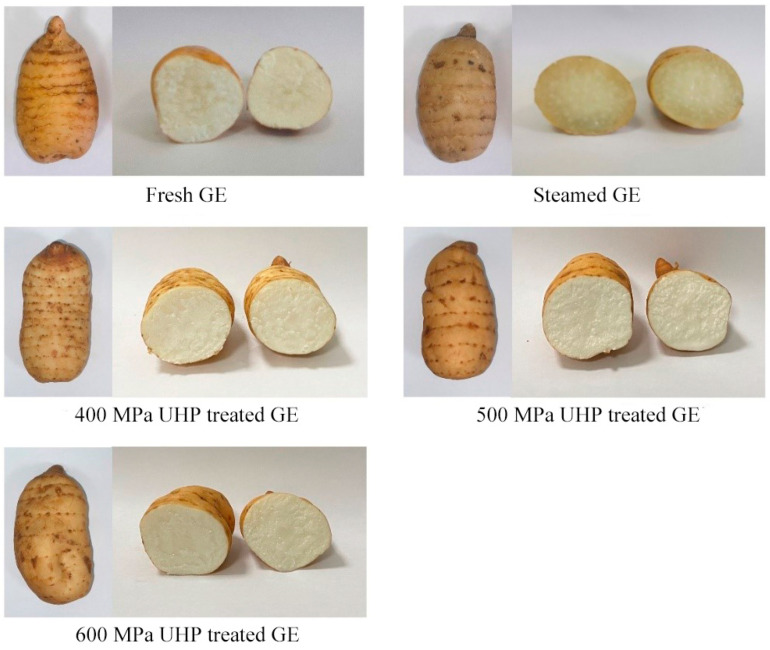
Effects of different treatment methods on the color of fresh GE.

As shown in Figure 1, compared to fresh GE, no differences in either the appearance or cross-sectional view of the samples treated with different pressure levels were observed. However, compared with the other samples, the cross-sectional color of steamed GE was glossy yellow. Thus, it can be concluded that compared to the traditional steaming pre-treatment, the applied UHP pre-treatment can retain the visual form of GE.

### 3.2. Water Detection with LF-NMR

#### 3.2.1. Drying Characteristics Analysis

**Figure 2:**
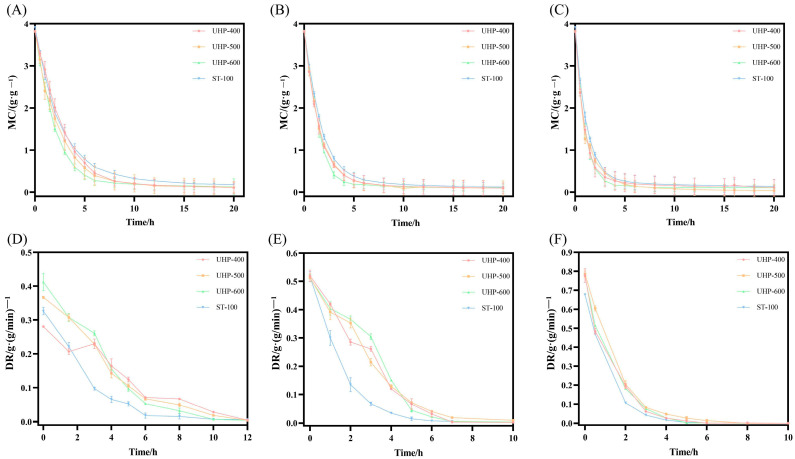
The MC and DR of GE samples at different temperatures: 40 °C (**A**,**D**), 60 °C (**B**,**E**), and 80 °C (**C**,**F**).

To determine the effect of UHP pre-treatment on the drying characteristics of GE, the pre-treated samples using UHP at different pressure conditions were dried by hot air at 40, 60, and 80 °C, and the developed drying curve and drying rate curve are illustrated in Figure 2. As shown in Figure 2, UHP pre-treatment at different pressures significantly affected the drying characteristics of the GE samples. As drying progressed, initially the *MC* of each sample was found to decrease rapidly and later become constant. Furthermore, during hot air drying at 40 °C, the steamed sample reached equilibrium (*MC* < 0.2) at 18 h, whereas the UHP-treated samples at 400, 500, and 600 MPa reached equilibrium at 12, 10, and 8 h, respectively. These results suggest that UHP pre-treatment can significantly reduce the time taken to reach drying equilibrium. Similarly, under hot-air drying at 60 °C, the steamed sample and UHP-pre-treated samples at 400, 500, and 600 MPa reached drying equilibrium at 10, 8, 6, and 6 h, respectively (Figure 2B). The drying equilibrium of the samples was reached in 6 h at a drying temperature of 80 °C (Figure 2C). In the early stage of drying (0–8 h), the *DR* gradually increased with an increase of UHP pre-treatment pressure. This could be due to the destruction of the tissue structure of GE by UHP and the diffusion of water in the GE samples [24]. In the later stage of drying, the GE samples shrank severely, resulting in a significant decrease in the surface area and a reduction in the water diffusion efficiency. Additionally, as shown in Figure 2D–F, with an increase in the drying temperature, the difference in the drying rates between the samples treated at different pressures and the steamed sample decreased. These results indicate that, in addition to the pre-treatment method, the drying temperature is another key factor that determines the drying rate.

**Figure 3:**
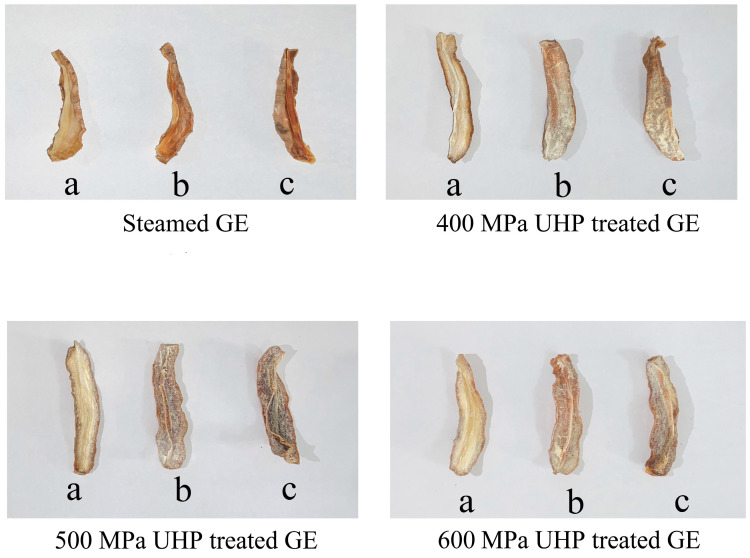
The appearance of GE samples under different treatment methods after hot-air drying at different temperature: 40 °C (a), 60 °C (b), and 80 °C (c).

The appearance of GE pre-treated under different UHP conditions followed by hot-air drying at different temperatures was studied, and the obtained results are illustrated in Figure 3. The results showed that, compared to steaming pre-treatment, UHP pre-treatment could alleviate the darkening appearance of GE samples to a certain extent. At the same time, since GE under hot-air drying had a better appearance with reduced energy consumption during the drying process, the drying temperature of 60 °C might be the best choice.

#### 3.2.2. LF-NMR Analysis

**Figure 4:**
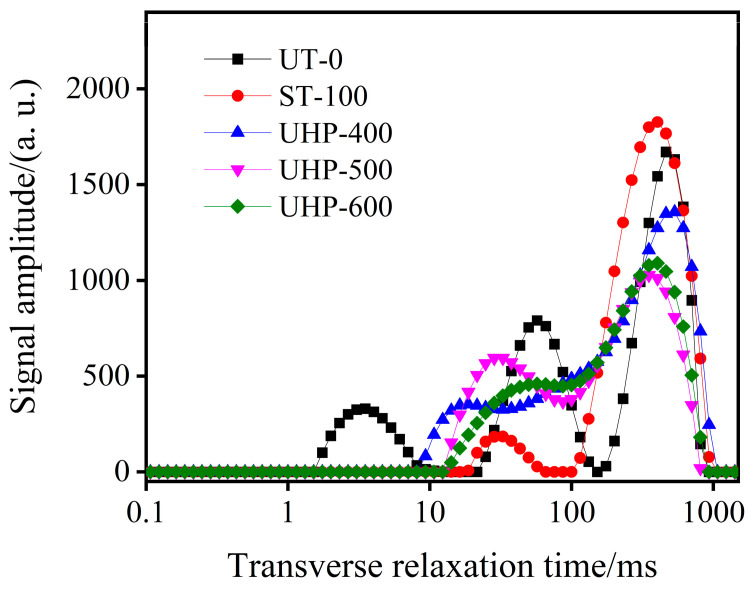
LF-NMR inversion of GE with different treatment methods.

After the drying process was conducted at 60 °C, LF-NMR analysis was performed separately for fresh GE, steamed GE, and UHP-treated GE groups, and the inversion curves were obtained (Figure 4). The locations of the relaxation peaks indicate the binding states of the hydrogen protons, whereas the ratio of each peak area to the total area reflects the proportion of water in that state relative to the overall water content. The alterations in the T2 curves, as illustrated in Figure 4, can be categorized into three distinct regions: T_21_ (0.01–10 ms), T_22_ (10–100 ms), and T_23_ (100–1000 ms), representing bound water, immobile water trapped in the cytoplasm, and free water, respectively. For fresh GE, the highest proportion was observed in free water (55.70%), followed by immobile water (30.59%), and bound water constituted the lowest proportion (13.71%). The GE samples subjected to steaming and UHP pre-treatment exhibited disappearance of the T21 peak. However, the T22 and T23 peaks increased, indicating improved fluidity of the water within the sample. Furthermore, the degrees of freedom associated with moisture are directly proportional to the drying rate (DR). Thus, both steaming and UHP pre-treatment methods can be considered beneficial for accelerating the drying process. This enhancement may result from the disruption of the cellular structure caused by the UHP or steam pre-treatment, which influences the interaction between bound water and macromolecules.

**Figure 5:**
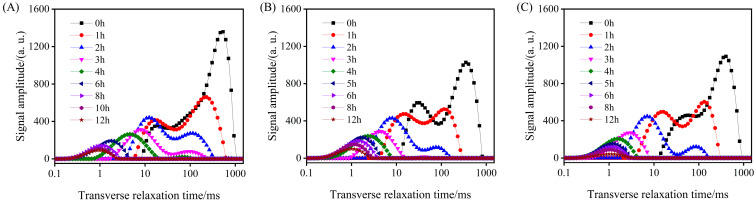
T2 relaxation spectra of GE samples under different UHP pre-treatment during hot-air drying at 60 °C: 400 MPa (**A**), 500 MPa (**B**), and 600 MPa (**C**).

Because the UHP pre-treatment could accelerate the drying process, the effect of different UHP pre-treatment pressures on the water state during hot air drying of GE at 60 °C was studied. As shown in Figure 5, as hot-air drying progressed, the relaxation peaks progressively moved to the left, while the areas of the peaks continuously decreased. Furthermore, the T_23_ peak decreased the most rapidly, indicating that the GE sample subjected to UHP pre-treatment primarily lost free water during the hot-air drying process. Furthermore, as the pressure of the UHP pre-treatment increased, the peaks shifted to the left more rapidly, and the areas of the relaxation peaks decreased at a faster rate.

**Figure 6:**
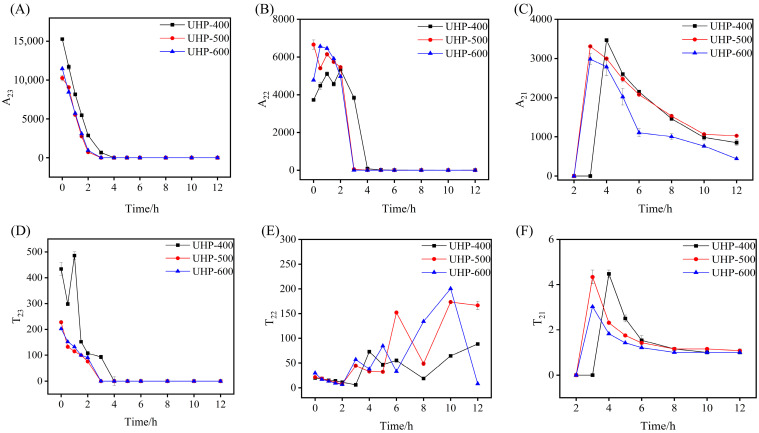
The influence curve of pressure on changes of migration of free water (**A**,**D**), immobilized water (**B**,**E**), and bound water (**C**,**F**).

To visually examine the alterations in water migration resulting from varying UHP pre-treatment pressures during the hot-air drying process of GE, the positions and peak areas of each relaxation peak were analyzed (Figure 6). T_21_, T_22_, T_23_, A_21_, A_22_, and A_23_ represent the positions and peak areas of the transverse relaxation peaks of free water, immovable water, and bound water, respectively, and *A_total_* represents the total peak area of each relaxation peak. As illustrated in Figure 6A, a decreasing trend in A_23_ was observed during hot-air drying. Furthermore, the rate of reduction in A_23_ increased progressively as the UHP pre-treatment pressure increased. This observed phenomenon may be attributed to the degradation of the GE tissue structure caused by the UHP pre-treatment. This degradation results in a progressive reduction in water constraints within the internal organization, which in turn leads to an increase in the degrees of freedom. Free water on the surface of the GE sample was removed, whereas the free water in the gaps rapidly diffused outward. As shown in Figure 6B, as the hot-air drying process progressed, the immobile water (A_22_) content of all samples initially increased and then started to decrease continuously. Furthermore, as the drying process progressed, the mobility of immobile water increased. As shown in Figure 6D, the peak position (T_22_) of immobile water exhibited an irregular change during drying. This could be attributed to tissue and cell membrane damage occurring during UHP pre-treatment, which results in the irregular movement of immobile water. Simultaneously, the free water converted into immobile water, which subsequently merges and contributes to the existing immobile water [25,26,27]. Furthermore, as shown in Figure 6C, no bound water was observed at the beginning of the drying process. However, with the continuous diffusion of both free and immobile water, a T_21_ peak appeared. The higher the UHP pre-treatment pressure, the earlier the bound water relaxation peak appeared in the sample. The drying process resulted in the migration of internal water towards regions of lower fluidity, causing the bound water to shift leftward, resulting in an increase in the peak area. At the conclusion of the 12 h drying period, the samples were found to contain only bound water.

**Figure 7:**
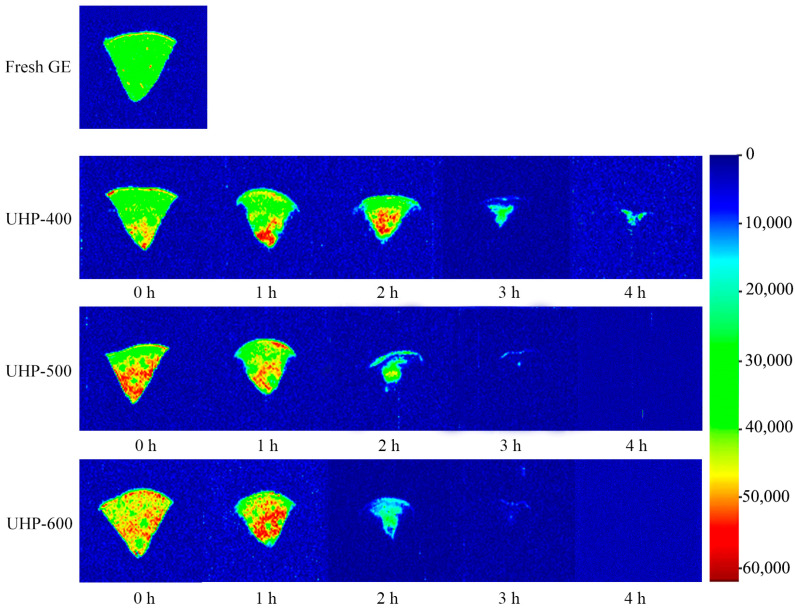
MRI images of GE samples under different UHP pre-treatment dried at 60 °C.

Low-field magnetic resonance imaging (LF-MRI) can be used to directly visualize the stage and distribution of water within a sample. As shown in Figure 3, 60 °C was selected as the best drying temperature, and the LF-MRI during the drying process of GE treated with various ultra-high-pressure treatments was analyzed. As illustrated in Figure 7, different colors correspond to different levels of hydrogen proton density; specifically, an increase in hydrogen proton density correlates with an increase in water content. Moisture was evenly distributed in the fresh GE sample. However, after UHP pre-treatment, water in the GE was concentrated inside the tuber, where the pseudo-color image is shown in red and yellow. As drying continued, the external water evaporated rapidly, creating a difference between the water content in the internal and external tissues, facilitating the continued movement of internal water towards the exterior. Notably, the higher the pressure in the UHP pre-treatment, the faster the water dissipation. After a drying period of two hours, the moisture content of the GE subjected to 500 and 600 MPa UHP pre-treatment was reduced, resulting in a predominance of blue to green regions. In contrast, the group that underwent 400 MPa UHP pre-treatment still exhibited residual red areas. Moreover, after further drying for 4 h at 60 °C, LF-MRI images of the 500 and 600 MPa UHP pre-treatment groups showed only a smaller amount of immobile water and bound water that was closely associated with the tissues. These results suggest that pre-treatment with 500 MPa UHP pre-t may be a good choice.

### 3.3. UHP Promotes the Retention of Active Ingredients in GE

The contents of six main compounds, namely gastrodin, p-hydroxybenzyl alcohol, parishin A, parishin B, parishin C, and parishin E in GE, were determined, and the results showed a good linear relationship (Table 1), R^2^ > 0.9992. The concentrations of the above-mentioned six compounds in the different GE treatments are summarized in Table 2. The concentration of p-hydroxybenzyl alcohol in the untreated GE group was the highest (5.92 mg/g). In contrast, the levels of gastrodin and parishins have been reported to be comparatively low. This could be due to the hydrolysis of ester or ether bonds found in gastrodin, from which parishins are hydrolyzed by *β*-D-glucosidase to produce p-hydroxybenzyl alcohol as the final product during the drying process. Both UHP and streaming pre-treatments have demonstrated efficacy in inhibiting parishins degradation in GE. Notably, compared with steaming pre-treatment, UHP pre-treatment was found to be capable of preserving more gastrodin, parishin A, parishin B, and parishin C. Furthermore, when compared to the steaming pre-treatment group, the p-hydroxybenzyl alcohol content in UHP-treated samples was reported to be lower, whereas that of gastrodin and parishins was reported to be higher. Notably, p-hydroxybenzyl alcohol can be obtained by breaking ester or ether bonds during GE steaming [9]. These results suggest that UHP pre-treatment can prevent the degradation of active substances due to high temperatures during drying.

### 3.4. UHP Reduces the Activity of Degrading Enzymes

**Figure 8:**
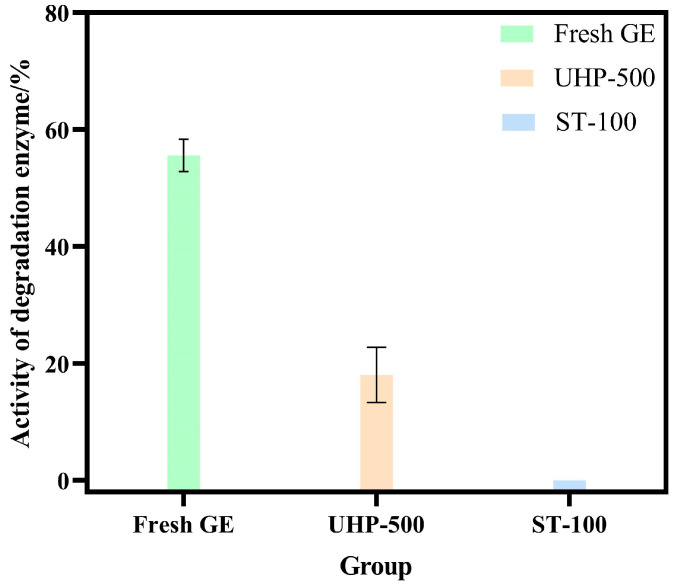
The activity of degradation enzyme in GE samples with different treatment methods.

The degradation of active ingredients in GE is mainly affected by the activity of degrading enzymes [9,28]. Thus, the activities of degradation enzymes in GE were analyzed. As shown in Figure 8, both UHP and steaming pre-treatments significantly reduced the activity of *β*-D-glucosidase. However, as per the results, the activity of *β*-D-glucosidase in GE is not completely inactivated, while in contrast, the steam treatment showed a stronger inactivation ability of the degrading enzyme. However, compared to the fresh GE group, the degradation of glycosides due to UHP pre-treatment was reported to be less, indicating that the application of UHP significantly enhanced the preservation of glycosides in GE by reducing the activity of the degrading enzymes.

### 3.5. UHP Enhances the Neuroprotective Activity of GE

**Figure 9:**
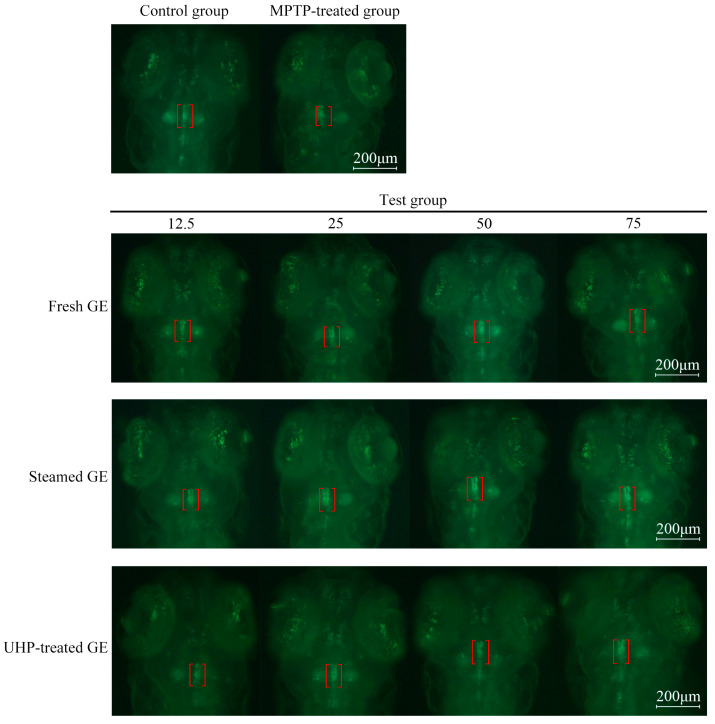
Effect of GE samples under different treatment methods on the transgenic Zebrafish.

**Figure 10:**
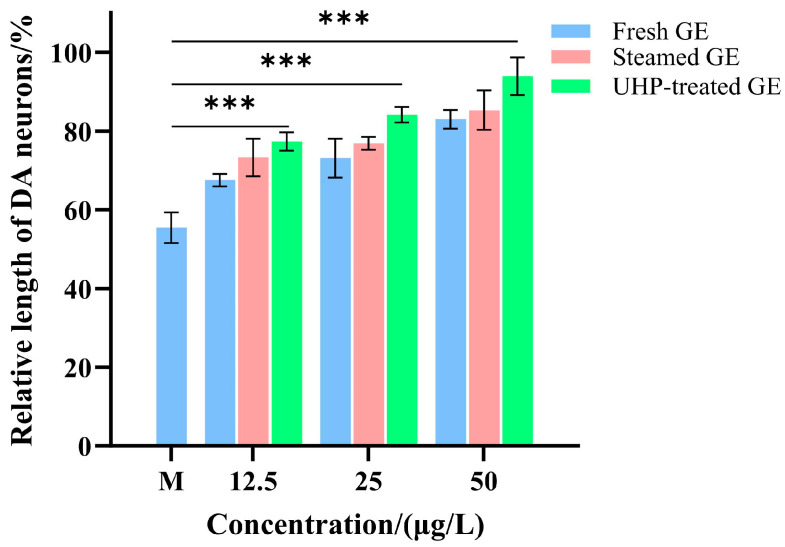
The neuroprotective activity of GE samples under different treatment methods. ***: Compared with M group, *p* < 0.001.

GE is commonly used to treat neurasthenia, vertigo, and headaches. In the present study, we evaluated the neuroprotective effect of GE. Representative fluorescence microscopy images of zebrafish across different treatment groups are shown in Figure 9, where the red brackets highlight the DA neurons in the zebrafish. The results showed that DA neurons in the control group developed fully, whereas in the MPTP-treated group, DA neurons in the larvae were noticeably absent and had significantly reduced length compared to those in the control group (*p* < 0.05). In contrast, a varying degree of increase in the length of DA neurons was observed in zebrafish treated with GE samples using different treatment processes. Further, as shown in Figure 10, with the increase of concentration of GE extract (12.5–75.0 μg/mL), a gradual increase in the length of DA neurons was observed with a dose-dependent trend. Compared to fresh GE, the UHP-treated GE extract increased the relative length of DA neurons. Furthermore, the extract of steamed GE demonstrated a significantly greater relative length of DA neurons compared to fresh GE (*p* < 0.001), suggesting that the processing method plays a crucial role in enhancing the bioactivity of GE. However, no significant difference was observed between the UHP and steamed pre-treatment groups (*p* > 0.05). At a concentration of 75.0 μg/mL, the relative lengths of DA neurons reached 93.39 ± 6.49% (UHP pre-treatment group) and 92.82 ± 2.41% (steamed pre-treatment group), respectively. These results suggest that both steaming and UHP pre-treatment enhanced the protection of DA neurons by GE in zebrafish and exhibited comparable activity. Therefore, UHP pre-treatment significantly improved the neuroprotective activity of GE by reducing the activity of degrading enzymes and increasing the content of active substances.

## 4. Conclusions

In this study, GE samples were treated with UHP technology and traditional steam technology, and compared to traditional steam treatment, UHP pre-treatment preserved the visual morphology of GE samples. In the hot-air drying experiment, the application of UHP pre-treatment enhanced the mobility of moisture, thereby decreasing the time required to reach drying equilibrium for the GE samples. Meanwhile, UHP pre-treatment reduced the activity of degradation enzymes and, combined with hot-air drying, enhanced the content of active substances and neuroprotective activity in vivo. Overall, UHP-assisted hot-air drying improved the quality of the GE samples.

## Figures and Tables

**Table 1:** Regression equation, linear range and regression coefficient of standard substances.

Compounds	Regression Equation	Regression Coefficient (R^2^)	Linear Range (mg/mL)
gastrodin	Y = 35,062X − 92.181	R^2^ = 0.9995	0.01~0.5
p-hydroxybenzyl alcohol	Y = 56,084X + 114.24	R^2^ = 0.9992	0.001~0.5
parishin E	Y = 22,813X − 74.322	R^2^ = 0.9998	0.01~0.25
parishin B	Y = 25,010X − 143.28	R^2^ = 0.9995	0.01~0.5
parishin C	Y = 36,166X − 115.64	R^2^ = 0.9997	0.01~0.5
parishin A	Y = 35,846X − 167.72	R^2^ = 0.9997	0.01~0.5

**Table 2:** The content of six active substances in GE samples with different treatment methods.

Treatment Method	The Content of Active Substances (mg/g)					
	Gastrodin	p-Hydroxybenzyl Alcohol	Parishin E	Parishin B	Parishin C	Parishin A
Fresh GE	0.31 ± 0.08	5.92 ± 0.44	1.11 ± 0.24	0.93 ± 0.26	0.70 ± 0.23	1.49 ± 0.44
Steamed GE	1.00 ± 0.15	1.92 ± 0.14	8.85 ± 2.67	2.99 ± 0.63	0.42 ± 0.09	5.24 ± 1.49
UHP-treated GE (500 MPa)	1.38 ± 0.16	0.75 ± 0.04	8.02 ± 1.71	8.07 ± 0.69	0.96 ± 0.08	7.21 ± 0.98

## Data Availability

The original contributions presented in the study are included in the article, further inquiries can be directed to the corresponding author.
